# Predictors of Diabetes Self-Management among Type 2 Diabetes Patients

**DOI:** 10.1155/2016/9158943

**Published:** 2016-08-03

**Authors:** Azylina Gunggu, Chang Ching Thon, Cheah Whye Lian

**Affiliations:** ^1^Department of Nursing, Faculty of Medicine and Health Sciences, Universiti Malaysia Sarawak, 94300 Kota Samarahan, Sarawak, Malaysia; ^2^Department of Community Medicine and Public Health, Faculty of Medicine and Health Sciences, Universiti Malaysia Sarawak, 94300 Kota Samarahan, Sarawak, Malaysia

## Abstract

Diabetes mellitus is a public health concern in Malaysia. Treatment of diabetes is costly and can lead to complications if disease is poorly controlled. Diabetes self-management (DSM) is found to be essential for optimal glycemic control. This cross-sectional study was conducted among samples from four randomly selected diabetes clinics in Sarawak, Malaysia. The aim was to determine the predictors for DSM. Face-to-face interview using questionnaire was used to collect data. Four hundred respondents with type 2 diabetes mellitus (T2DM) were recruited. Majority of the respondents were Sarawak Bumiputra (Iban and Bidayuh, 48.6%) and female (68.6%). The mean age was 58.77 years (SD = 11.46) and approximately half of the respondents (50.6%) had T2DM for six years (SD = 4.46). The mean fasting blood glucose (FBG) was 8.06 mmol/L (SD = 2.94), with majority (76.1%) having the level higher than 6.1 mmol/L. Multiple logistic regression tests showed significant linear relationship between DSM and belief in treatment effectiveness (*p* = 0.001), family support (*p* = 0.007), and self-efficacy (*p* = 0.027). Health care personnel must convince patients with T2DM of the effectiveness of the treatment, empower and enhance their self-efficacy, and enlist the family support so as to ensure patients sustain their DSM efforts.

## 1. Introduction

As Malaysia progresses both socially and economically, noncommunicable diseases have also fast become its public health concern. One of these diseases is diabetes mellitus (DM). Malaysia burden of DM continues to increase between 2009 and 2012; Malaysia National Diabetes Registry registered a total of 653,326 patients diagnosed with T2DM. The prevalence of diabetes in Malaysia is projected to be 21.6% of its adult population by the year 2020 [1]. DM has been shown to be closely related to increased premature and preventable mortality, as well as macro- and microvascular complications such as heart disease, stroke, end-stage renal failure, blindness, and amputation. It is also very costly to treat patients with DM. A study in Malaysia showed that Ministry of Health Malaysia spent a calculated amount of RM386,531.21 for a 6-month period to manage DM [2]. The same study showed that estimated direct cost per patient was RM2,684.24 and for indirect cost RM1,062.88 annually.

To reduce the complications of DM and in turn the cost of treatment, it is important for patient to achieve good glycemic control. Good glycemic control can be measured by HbA_1c_ test. A study found that a 1% reduction in HbA_1c_ was associated with a 37% decrease in risk for microvascular complications and a 21% decrease in the risk of death related to diabetes [3]. Evidence from many previous studies shows that self-management training in T2DM is effective for short-term glycemic control [4]. Another study found that adherence to self-management is crucial in the overall management of diabetes and those who perform diabetes self-management (DSM) effectively achieve better short- and long-term health [5].

Sarawak, one of the states in East Malaysia, registered a total of 43,333 patients with T2DM during the period of 2009 to 2012 [1]. Out of those who had HbA_1c_ test, 39.1% achieved the Malaysian glycemic treatment target of HbA_1c_ <6.5%. The percentage of those who achieved the glycemic treatment target would even be lower if those who did not have HbA_1c_ were included. Thus, it is important to ascertain the reasons for low percentage of patients achieving glycemic control in order to plan and implement effective interventions. This study aimed to identify the predictors for DSM. This paper is based on a master thesis done by Gunggu [6].

## 2. Materials and Methods

This was a cross-sectional study conducted in four randomly selected diabetes clinics in Kuching and Samarahan Division of Sarawak. Systematic random sampling method was used to recruit respondents. The inclusion criteria were patients (a) with T2DM for more than one-year duration, (b) aged 18 years to 65 years, (c) able to understand English or Bahasa Melayu, and (d) resident in the two divisions for at least six months. Those with sight problem and cognitively challenged were excluded from the study. Sample size was determined using the formula by Naing et al. [7], where *n* = *Z* ^2^ *P*  (1 − *P*)/*d* ^2^. Based on the mean prevalence of good control of 38.9% [8], *P* was determined at 0.389. *d* was set as +0.05, and the level of statistical significance, *α*, was 0.05. A 10% of attrition rate was added to determine a sample size of 400. Ethical approval was obtained from Research and Ethics Committee, Universiti Malaysia Sarawak, and Medical Research and Ethics Committee, Ministry of Health, Malaysia (NMRR-12-5-10829). All respondents signed an informed consent.

Data were collected via a face-to-face interview to ensure consistency as majority of the respondents were illiterate. The questionnaire consisted of seven sections. Section A was designed to obtain demographic data and the health profile of respondents (10 items). Section B consisted of 10 items assessing DSM. There were five behaviours involved: diet habit, exercise, medications compliance, self-monitoring of blood glucose, and foot care. Two items—performance of insulin injection and self-monitoring of blood glucose (2 items)—were excluded in this study. This decision was made based on previous study that showed that only 12.9% of the Malaysian diabetes patients were on insulin therapy and only 3.4% of patients with T2DM performed self-monitoring of blood glucose [9]. To respond, respondents were asked to recall their activities for the last seven days and stated the numbers of days they performed the DSM. DSM was calculated based on the summed numbers of days. A higher score indicates a higher level of self-care management.

Section C of the questionnaire collected data on “beliefs in treatment effectiveness” which consisted of nine items. The questionnaire assessed two main aspects: (a) the belief that DSM activities were important in controlling the blood glucose—items 1 to 4—and (b) the belief that DSM activities were important in preventing the diabetes-related complications. Five-point Likert-type scale was used to score the items. For items 1 to 4, 0 indicated “not important,” while 4 indicated “extremely important.” Items 5 to 9 asked the respondent's belief in the DSM activities in preventing the diabetes complications; the 5-point Likert scale was from 0 (not possible) to 4 (extremely possible). Higher score denoted greater perceived beliefs in the treatment effectiveness in controlling the illness and preventing the complications.

Section D of the questionnaire assessed the respondents' level of self-efficacy. It comprised seven items, and 5-point Likert scale was used for the measurement with lowest score of 0 (definitely yes) to 4 (definitely not), the highest. In this part, respondents were asked how they perceived their capability in performing the DSM activities: diet, exercise, monitoring their blood glucose, foot care, and taking medication. Lower scores demonstrated the higher confidence level in performing DSM activities.

Section E has seven items measuring the perceived support received by the respondents' in the past three months. The perceived support was measured according to the 5-point Likert scale, from 0 (never) to 4 (always). Higher scores demonstrated better family support as perceived by the respondent. Section F has seven items that collected data on the healthcare team provider-patient communication. In this study, the word “doctor” from the original questionnaire was changed to “healthcare provider” because in local setting, diabetes patients were assessed by either the assistant medical officer or nurses during the follow-up visits. Patients are referred to doctor only if there are complications. Besides, the term “healthcare provider” provided a wider scope for respondents when reflected on their communication during follow-up. A 5-point Likert scale from 0 (never) to 4 (always) was used. Higher scores indicated better healthcare provider and patient's communication. Questionnaire for Sections B, C, D, E, and F was adopted with permission from Xu et al. [10].

Section G assessed respondents' health literacy on diabetes. Health literacy on diabetes meant the respondents' understanding of information in relation to diabetes and its management. A Malay language version of the questionnaire was adapted from Gazmararian et al. [11] with permission. It consisted of 11 items with true-false response. A higher score indicated higher knowledge on diabetes mellitus. All items in the questionnaire that were in English were translated to Bahasa Melayu version using back to back translation. A pilot study was conducted for the items in Sections B, C, D, E, and F and the Cronbach's alpha for the questionnaire ranged from 0.537 to 0.873. Although deleting one of the items could improve the Cronbach's alpha to 0.563, it was decided to retain that item. As this item assessed the oral medication adherence, deleting it would lead to incomplete assessment of DSM, as taking medication was an important regime in DSM in this study. The data were analysed using Statistical Package for Social Sciences (SPSS) version 20.

## 3. Results

A total of 400 respondents were recruited with a mean age of 58.77 (SD = 11.46). Majority of the respondents were women (68.6%) and 48.6% were Sarawak Bumiputra (Iban and Bidayuh). About 84.5% of them were married and 38.4% had no formal education. Approximately half (50.6%) of the respondents had diabetes mellitus for less than five years with a mean of 6.40 years (SD = 4.46). Most of the respondents (84.8%) had one or two chronic illnesses with the commonest being hypertension and hyperlipidemia. A total of 19.6% of them reported to have diabetes-related complications such as neuropathy, nephropathy, retinopathy, and heart problems. The mean fasting blood glucose (FBG) was 8.06 mmol/L (SD = 2.94), in which majority (76.1%) had their FBG more than 6.1 mmol/L. Of those with reported HbA_1c_ results (*n* = 137), 56 respondents (40.9%) had their HbA_1c_ equal to or more than 6.5%. Majority of respondents (79.1%) were not treated with insulin therapy. Detailed information on demographic characteristic and health profile of the respondents is presented in Table 1.

Table 2 shows the total mean score of the DSM and the five factors that might influence the DSM behaviours: “belief in treatment effectiveness,” “self-efficacy,” “family support,” “healthcare provider-patient communication,” and “diabetes-related knowledge.” The total mean score for DSM was 29.97 (±7.53). The mean score for each factor varied from 8.31 (±1.82) to 26.79 (±4.60).

Majority of the respondents reported that they took their oral antidiabetic agents according to the doctor's prescription (84.0%) and controlled their diet (60.8%) every day for the seven days prior to the data collection date, while 86.3% reported that they took their meal at a regular time every day. In terms of physical activity, only 29.1% of the respondents reported that they participated in physical activity for at least five days in the past week. However, lower mean of 0.67 ± 1.44 was shown in relation to the engagement with specific exercise. Three hundred and twelve (78.0%) respondents reported that they dried in between their toes after washing their feet, while 136 (34.0%) reported that they performed foot inspection every day.  Table 3 shows the details.

The predictors of DSM were evaluated using regression analyses. Single linear regression analysis showed that none of the sociodemographic characteristics had significant relationship with DSM. Multiple linear regression analysis showed significant linear relationship between DSM and belief in treatment effectiveness (*p* = 0.001), family support (*p* = 0.007), and self-efficacy (*p* = 0.027) (Table 4). For one unit, increment of the respondent's belief in treatment effectiveness would cause 0.301-unit (95% CI: 0.13, 0.47, *p* = 0.001) increase in the level of DSM and DSM increased 0.198 times in a unit of family support increment (95% CI: 0.05, 0.34, *p* = 0.007). Those type 2 diabetes patients with a unit more in self-efficacy level have 0.210 units higher in DSM performance (95% CI: 0.024, 0.395, *p* = 0.027). From these findings, three variables predicted DSM. The relations among all the predictors in this study can be further described based on the generic regression equation:

(1) $$\begin{array}{c} Y=a+b1X1+b2X2+\cdots \end{array}$$

in which *Y* indicate the dependent variable, *a* is the intercept, and *X* is the independent variables whereas *b* is the regression coefficient [12].

Thus, the regression equation (final model) was DSM = 12.02 + 0.301(BTE) + 0.198(FS) + 0.210(SE). *R* for the regression model was significant, *F*(4, 395) = 14.59, with *p* = 0.000. *R* ^2^ of 0.129 indicated that this model accounts for about 12.9% of the variance in DSM.

## 4. Discussions

DSM was assessed based on four behaviours performed by individuals with DM. It involved the practice of diet control, engaging with adequate physical activities, taking medications, and practicing foot care. Performing self-monitoring of blood glucose was not assessed in this study compared to the previous study [10] as it was not a common practice among the individuals with T2DM as majority of them were treated with oral antidiabetic agents in Malaysia [9]. Performing good DSM is essential for individuals diagnosed with DM, as it is the key in diabetes management to ensure good control of serum glucose, thus, preventing the occurrence of diabetes-related complications [5].

More than 80% (84.2%) of the respondents in this study reported that they took their oral antidiabetic agents everyday according to doctor's prescription. This high percentage of medication compliance was also found in China (89.1%) [10]; however studies in the US population showed that only 64% of their patients did so [10]. This high percentage of medication compliance compared to other behaviours in DSM in this study could indicate that most patients with T2DM preferred taking medications rather than modifying their behaviour, which is always more difficult. Individuals who have better adherence to medications are believed to be significantly associated with positive attitudes towards DSM [13].

Findings in the current study indicated that lower percentage of respondents practiced diet control (60.8%). Personal unwillingness and numbers of social gatherings as well as time and energy needed for food preparations were some of the barriers highlighted in previous studies that hindered compliance to diabetic diet [14, 15]. Previous study had shown that proper counselling on diet control would lead respondents to have better understanding of its importance, resulting in them having significant reduction of total HbA_1c_ level and BMI [16].

In terms of physical activity, only 29.1% of the respondents engaged in 30-minute physical activities for at least five days a week. In a study conducted in the United States, a slightly higher percentage (35%) of exercise prevalence was reported [17]. The barriers to perform physical activity among patients with T2DM could be related to bad weather (hot or rainy day), staying at housing area (lack of available walking area), and busy routine. Age could be another possible contributing factor to this poor performance, taking into account that almost half (45.4%) of the respondents were those aged more than 60 years, who might not be able to perform regular exercise as recommended due to poor health. Inadequate or low exercise performance by respondents could also be related to culture. Unlike Chinese or Western population, neither Malay nor Sarawak Bumiputra (the Iban or Bidayuh) population have specific physical activities such as yoga and Tai Chi which are culturally related. Some studies showed that the Chinese tended to exercise more and were more health conscious [18].

Proper foot care is essential in preventing complications such as foot ulcer and limb amputation [19]. However, this study found that only 34% of the respondents performed daily foot inspection. Previous study [17] showed higher proportion (63%) of diabetes patients practicing daily foot care. One of the possible explanations for such difference could be due to lack of knowledge in relation to the foot care. Studies had shown that educational intervention on proper foot care had resulted in better self-foot-care behaviour, improved knowledge, and increased level of confidence in terms of DSM [20]. Evidence also showed that providing foot care education to diabetes patients increased their awareness, resulting in low incidence of foot amputation [21].

This study also found that those who had spouse were significantly performing better DSM compared to those who were single, widowed, or divorced. Involvement of spouse in self-management of the disease may include providing social support to affirm healthy lifestyle behaviour as well as social control to modify their health behaviour [22]. Beverly et al. [23] found that spousal support was associated with long-term adherence to major lifestyle changes, suggesting that patients with T2DM were able to obtain emotional and physical support that aided in weight loss, proper diabetic diet, and adherence to the follow-up. This in turn would result in better diabetic control and reduce the risk of complications.

Self-efficacy was found to be a predictor for DSM. Individuals who had chronic illness (diabetes, hypertension, and arthritis) were able to demonstrate better levels of adherence to their self-care if they possessed higher level of self-efficacy [24]. Other studies revealed that individuals with higher level of self-efficacy were more likely to present better metabolic control as it was a predictor for diet management, self-care, and engagement in physical activities [17, 25]. As such, health care professionals should design strategy to enhance patients' self-efficacy. Anderson and Funnell [26] suggested empowerment approach to promote patients' level of confidence which enhances their ability to think critically and act autonomously and improves the level of efficacy. Tang et al. [5] showed that for those patients who had undergone two-year empowerment-based DSM interventions had significant improvements in adherence to the treatment regimens.

This study also found belief in treatment effectiveness predictive of better DSM which is consistent with previous studies [27]. As more than 80% of the respondents in this study reported that they took their oral antidiabetic agents everyday according to doctor's prescription, it is congruent to find belief in treatment effectiveness as one of the predictors for DSM.

Consistent with previous studies [9], family support was found to be a predictor for adherence to DSM. Strong support from family builds patients' confidence level, resulting in effective self-management and better disease control [10]. Social support can have the appraising and informative effects and provide coping strategies to assist patients to manage diabetes-associated stress and altered daily routines [28]. Mayberry and Osborn [29] found that family members who showed better supportive behaviours were those who were more knowledgeable about diabetes. Thus, in clinical intervention it is important to enhance better understanding of disease and interactions between patients and their family members so as to promote positive and supportive behaviours. Hence, DSM should be a joint effort of the patients and their family.

This study was conducted in two divisions of Sarawak and majority of the respondents were Malays and Sarawak Bumputra; thus, the results may not be generalizable to all the populations of Malaysia.

## 5. Conclusions

This study added knowledge to the list of predictors for DSM especially for patients with T2DM in Malaysia. As the predictors found are belief in treatment effectiveness, family support, and self-efficacy, health care personnel should convince patients with T2DM of the effectiveness of the treatment. They also need to empower the patients to enhance their self-efficacy and enlist the support from their family to ensure that patients could sustain their self-management efforts.

## Figures and Tables

**Table 1:** Sociodemographic characteristics and health profile of the respondents (*n* = 400).

Demographic characteristics/health profiles	Respondents		
	Frequency	Percentage	Mean ± SD
*Age*			58.77 ± 11.46
*Gender*			
Male	126	31.4	
Female	274	68.6	
*Race*			
Malay	131	32.7	
Iban & Bidayuh	195	48.8	
Chinese & others	74	18.5	
*Marital status*			
Having spouse	338	84.5	
Without spouse	62	15.5	
*Education level*			
No education	154	38.4	
Primary education	171	42.9	
Secondary education and higher	75	18.7	

*Duration of having diabetes mellitus*			6.4 ± 4.47
5 years and below	202	50.6	
More than 5 years	198	49.4	
*With chronic illness*			
None	49	12.2	
1-2	339	84.8	
3 and above	12	3.0	
*Diabetes-related complications*			
None	333	83.3	
1-2	67	16.7	
*Latest HbA*_*1c*_ (*n* = 137)			6.67 ± 2.48
Less than 6.5%	81	59.1	
6.5% and above	55	40.9	
*Current fasting blood sugar*			8.06 ± 2.94
Less than 4.4 mmol/L	9	2.2	
4.4–6.1 mmol/L	87	21.7	
More than 6.1 mmol/L	304	76.1	
*Treatment *			
Without insulin	317	79.1	
With insulin	83	20.9	

**Table 2:** Mean and standard deviation of the DSM and factors that may influence DSM behaviours (*n* = 400).

Variable	Mean (SD)	Min.	Max.
DSM^#^	29.97 (7.53)	9	49
Belief in treatment effectiveness	26.79 (4.40)	15	36
Self-efficacy	19.93 (3.86)	3	27
Family support	12.06 (5.42)	0	24
Healthcare provider-patient communication	21.43 (3.74)	11	28
Knowledge	8.31 (1.82)	3	11

^#^DSM: diabetes self-management.

**Table 3:** DSM status of the respondents (*n* = 400).

Item number	DSM behaviour	Respondents		
		Number	Percentage	Mean ± SD
1	*Taking oral medication*			6.61 ± 1.22
	Following doctor's prescription	336	84.0	
	Missed at least once	64	16.0	
2	*Followed diabetic diet*			5.47 ± 2.33
	Everyday	243	60.8	
	Missed at least a day	57	39.2	
3	*Regular meal time*			6.59 ± 1.14
	Everyday	345	86.3	
	Missed at least a day	55	13.7	
4	*Physical activity for at least 30 minutes*			2.44 ± 2.65
	At least five days	116	29.1	
	Less than five days	284	70.9	
5	*Doing specific exercise*			0.67 ± 1.44
	At least five days	16	4.1	
	Less than five days	384	95.9	
6	*Checking foot*			2.62 ± 3.27
	Everyday	136	34.0	
	Missed at least a day	264	66.0	
7	*Drying foot*			5.53 ± 2.82
	Everyday	312	78.0	
	Missed at least a day	88	22.0	

**Table 4:** Factors predicting the DSM.

Variables	SLR^a^		MLR^b^		
	*b* ^c^ (95% CI)	*p* value	Adj. *b* ^d^ (95% CI)	*t*-stat	*p* value
Constant			12.02 (6.68, 17.37)		
*Sociodemographic & treatment option*					
Age (years)	−0.01 (−0.08, 0.04)	0.614	—	—	—
Gender	0.26 (−1.33, 1.86)	0.744	—	—	—
Marital status	−0.53 (−1.76, 0.70)	0.399	—	—	—
Years of education	0.00 (−0.17, 0.18)	0.934	—	—	—
*Health profile*					
Duration of DM	−0.06 (−0.22, 0.10)	0.475	—	—	—
Chronic illness	−0.92 (−3.18, 1.33)	0.421	—	—	—
Complication	−1.69 (−3.67, 0.28)	0.092	—	—	—
*Others predicting factors*					
Belief in treatment effectiveness	0.47 (0.31, 0.62)	0.000^*∗*^	0.301 (0.13, 0.47)	3.46	0.001^*∗*^
Family support	0.35 (0.22, 0.48)	0.000^*∗*^	0.198 (0.05, 0.34)	2.71	0.007^*∗*^
Healthcare provider-patient communication	0.19 (−0.00, 0.38)	0.058	—	—	—
Knowledge	0.86 (0.47, 1.26)	0.000^*∗*^	0.400 (−0.01, 0.81)	1.92	0.055
Self-efficacy	0.337 (0.14, 0.52)	0.001^*∗*^	0.210 (0.02, 0.39)	2.22	0.027^*∗*^

^a^Simple linear regression.

^b^Multiple linear regression (*R* ^2^ = 0.129); the model reasonably fits well; model assumptions are met: there is no interaction between independent variables and multicollinearity problem.

^c^Crude regression coefficient.

^d^Adjusted regression coefficient.

^*∗*^ *p* < 0.05.
